# Choroidal melanoma metastatizing to the biliary system: A diagnostic dilemma

**DOI:** 10.4103/0971-5851.65337

**Published:** 2009

**Authors:** Samir R. Agarwal, Indranil Bhattacharya, Yoganand V. Patil, Anjali D. Amrapurkar

**Affiliations:** *Department of Pathology, Jagjivanram Hospital, Western Railway, Maratha Mandir Marg, Mumbai Central, Mumbai – 400 008, India*

**Keywords:** *Choroidal melanoma*, *gall bladder*, *metastases*

## Abstract

Metastatic melanoma to the gall bladder is extremely rare and is associated with a very poor prognosis. We report a case of choroidal melanoma metastatizing to the hepatobiliary system, with an unusual presentation. Our patient presenting with obstructive jaundice was misdiagnosed as having carcinoma of the gall-bladder, but the diagnosis of metastatic melanoma to the gallbladder was confirmed by ultrasonography guided fine needle aspiration cytology (USG-FNAC). On reviewing the past history, the patient had a history of enucleation for choroidal melanoma. Even though the liver ‘is’ a common site for metastasis of choroid melanoma, the patient presenting with a suspected gall bladder mass ‘is’ a rare presentation. Hence, gastrointestinal symptoms and a history of melanoma should be investigated for the presence of gastrointestinal or liver metastases, even if the original primary malignancy was diagnosed years before the patient‘s presentation.

## INTRODUCTION

Choroidal melanoma is the most common, primary, intraocular, malignant tumor and the second most common type of primary malignant melanoma in the body.[1] It is nevertheless an infrequently found tumor. The principal target organ for metastasis of choroidal melanoma is the liver, with almost 50% of the patients developing liver metastases up to 15 years after diagnosis.[2] Metastatic melanoma to the gall bladder is extremely rare and is associated with a very poor prognosis.[3]

## CASE REPORT

A 38-year-old male presented with complaints of pain in the abdomen with heaviness and symptoms of obstructive jaundice. The patient was diagnosed as a case of carcinoma-gallbladder with liver metastasis on the basis of the findings from the computed tomography of the abdomen (CT-abdomen).

**Figure 1:**
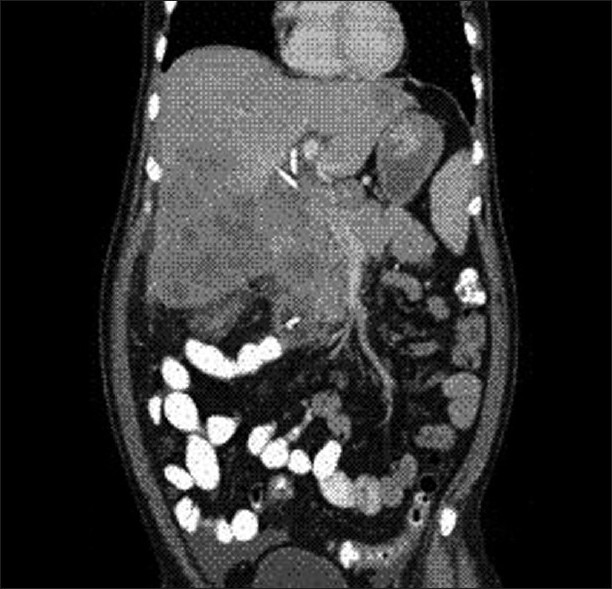
CT abdomen (coronal section) showing a large caudate lobe liver mass involving the gall bladder and common bile duct at porta with intrahepatic biliary radical (IHBR) dilatation

**Figure 2:**
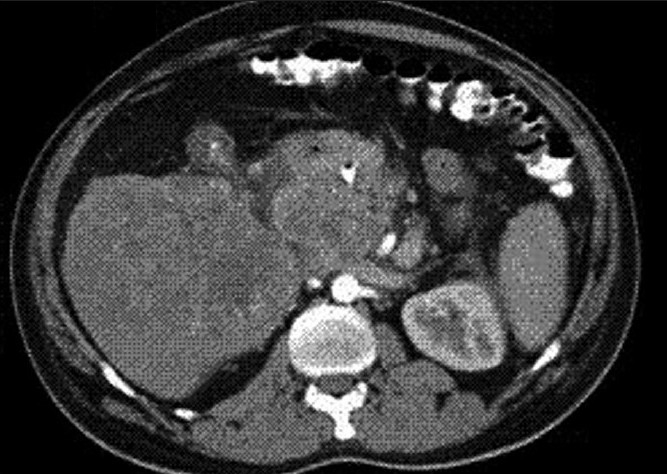
CT abdomen (Transverse section) showing a large caudate lobe liver mass involving the gall bladder and common bile duct at porta with IHBR dilatation

Physical examination revealed severe icterus and liver enlargement up to 5 cm below the right costal margin. The liver was hard and nontender. Ultrasonography (USG) and computed tomography (CT scan) revealed a large right and caudate lobe liver mass involving the gallbladder and common bile duct, with intrahepatic biliary radical (IHBR) dilatation, favoring the diagnosis of carcinoma gall-bladder with liver metastasis [Figures 1 and 2].

**Figure 3:**
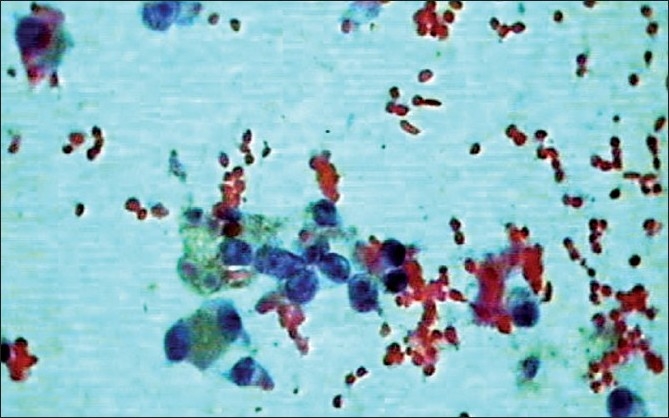
Photomicrograph showing polyhedral cells arranged singly and in loose aggregates possessing a moderate amount of cytoplasm containing brown pigment (Papanicolaou stain, ×400)

Ultrasonography guided fine needle aspiration of the lesion revealed polyhedral cells arranged singly and in loose aggregates possessing a moderate amount of cytoplasm containing brown pigment and eccentrically placed enlarged vesicular nuclei, with prominent eosinophilic nucleoli [Figure 3]. These features were suggestive of a metastatic malignant melanoma.

**Figure 4:**
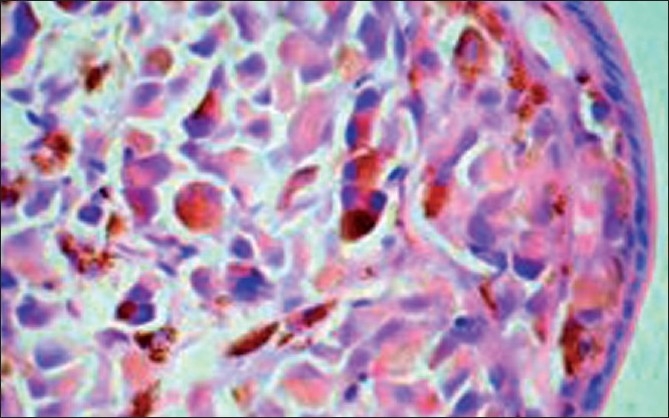
Photomicrograph showing pigmented tumor cell infiltration subepithelially and in the lamina propia of the duodenum (H and E, ×400)

A detailed history retrospectively revealed a past history of choroidal melanoma for which enucleation was done three years prior. Subsequently, the patient underwent upper gastrointestinal endoscopy, which revealed black colored nodules in the second part of the duodenum. A targeted biopsy from the lesion showed features of malignant melanoma [Figure 4]. The tumor cells were positive for Masson-Fontana silver stain.

## DISCUSSION

Intraocular malignant melanoma originates from uveal melanocytes in the uveal stroma, and is the most common primary intraocular tumor in adults.[2] The choroid is the layer between the retina and the sclera. The choroid is considered part of the uveal tract composed of the iris and ciliary body anteriorly and the choroid posteriorly. Intraocular melanomas can simultaneously involve more than one uveal structure.

The yearly incidence of choroidal melanoma in the US is six to seven cases per million people. Sixty-five percent of melanoma patients are over the age of 50.[4] Choroidal melanoma is more common in whites than in other races. Whites are eight times more likely to have melanoma than African-Americans and three times more likely than Asians.[5]

Choroidal melanomas ultimately cause death, practically always secondary to distant metastases rather than a local spread, the principal target organ for metastasis is the liver followed by lungs, skin, and bone. It can only spread hematogenously, because there are no lymphatic vessels in the eye.

Metastatic liver disease is the overwhelming cause of death in uveal melanoma patients, with almost 50% of the patients developing liver metastases up to 15 years after diagnosis. Most of these patients do not present with any evidence of overt metastasis at the time of initial diagnosis although it is assumed that they have undetectable micrometastases.[2] Metastatic melanoma to the gall bladder is extremely rare and is associated with a very poor prognosis.[3]

Autopsy series have demonstrated that metastasis to the gallbladder may occur in 4 to 20% of the patients with melanoma. Despite this statistic, it is rare for metastatic melanoma involving the gallbladder to cause symptoms during life.[6] As a result of the rarity of gallbladder melanoma cases, the diagnosis is often not suspected preoperatively.[7] Ultrasonic features may help to diagnose melanoma metastases preoperatively. Metastatic melanoma of the common bile duct (CBD) is very rare, with only 18 cases reported so far;[8] the most common presentation in these cases is of progressive painless obstructive jaundice with few rare cases presenting with hematobilia,[9] pain,[10] or cholangitis.[11] Laboratory investigations in these cases suggest cholestatic jaundice.

The first case of a metastatic melanoma to the bile duct was described by Spigelberg in 1895, and the second by Duval in 1908.[12] The melanoma secondaries usually originate from the primary skin lesion, but occasionally, may originate from the primary or metastatic melanoma of the gall bladder. Patients with metastatic melanoma to the CBD usually present with progressive painless obstructive jaundice. Obstructive jaundice as the first symptom of the disease due to metastatic melanoma causing ampullary obstruction has been reported only once.[13] Our patient presented with a similar picture of obstructive jaundice due to tumor extension into the bile duct and involvement of the ampulla.
